# Supplementary material for: Correlation of membrane protein conformational and functional dynamics
Source: Nat Commun. 2021 Jul 16;12:4363. doi: 10.1038/s41467-021-24660-1 (PMC8285522; doi:10.1038/s41467-021-24660-1)
Supplement: Supplementary file 1 — Supplementary Information [file 41467_2021_24660_MOESM1_ESM.pdf]

Correlation of Membrane Protein Conformational and Functional Dynamics

Raghavendar Reddy Sanganna Gari[1,2], Joel José Montalvo-Acosta[3], George R Heath[1,4], Yining Jiang[2], Xiaolong Gao[1], Crina M Nimigean[1,2], Christophe Chipot[3,5,]* and Simon Scheuring[1,2,]*

[1] Weill Cornell Medicine, Department of Anesthesiology, 1300 York Avenue, New York, NY-10065, USA.
[2] Weill Cornell Medicine, Department of Physiology and Biophysics, 1300 York Avenue, New York, NY-10065, USA.
[3] Laboratoire International Associé CNRS and University of Illinois at Urbana-Champaign, F-54506 Vandœuvre-lès-Nancy, France.
[4] current affiliation: Astbury Centre for Structural Molecular Biology, School of Physics & Astronomy, University of Leeds, Leeds, UK
[5]Department of Physics, University of Illinois at Urbana-Champaign, Urbana, Illinois 61801, USA.

* Correspondence to: sis2019@med.cornell.edu

Supplementary Figures

Supplementary Figure 1:

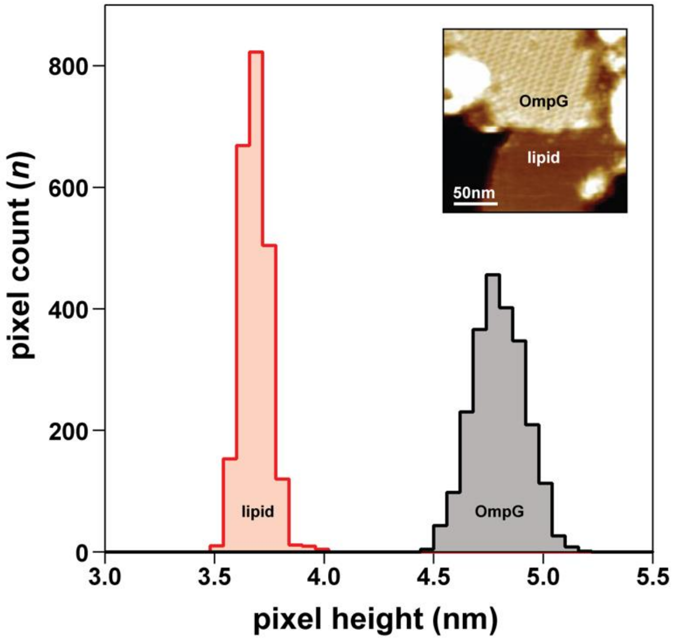

Figure S1) Height of OmpG molecules in a lipid bilayer. Histogram of heights of pixels of lipid bilayer (red, 3.7 nm) and OmpG 2D lattice (black, 4.8 nm), where the mica sample support is set to 0.0nm. Inset shows HS-AFM image of a membrane and densely packed OmpG molecules. These height measurements are consistent over multiple membranes from multiple samples over several imaging days.

Supplementary Figure 2:

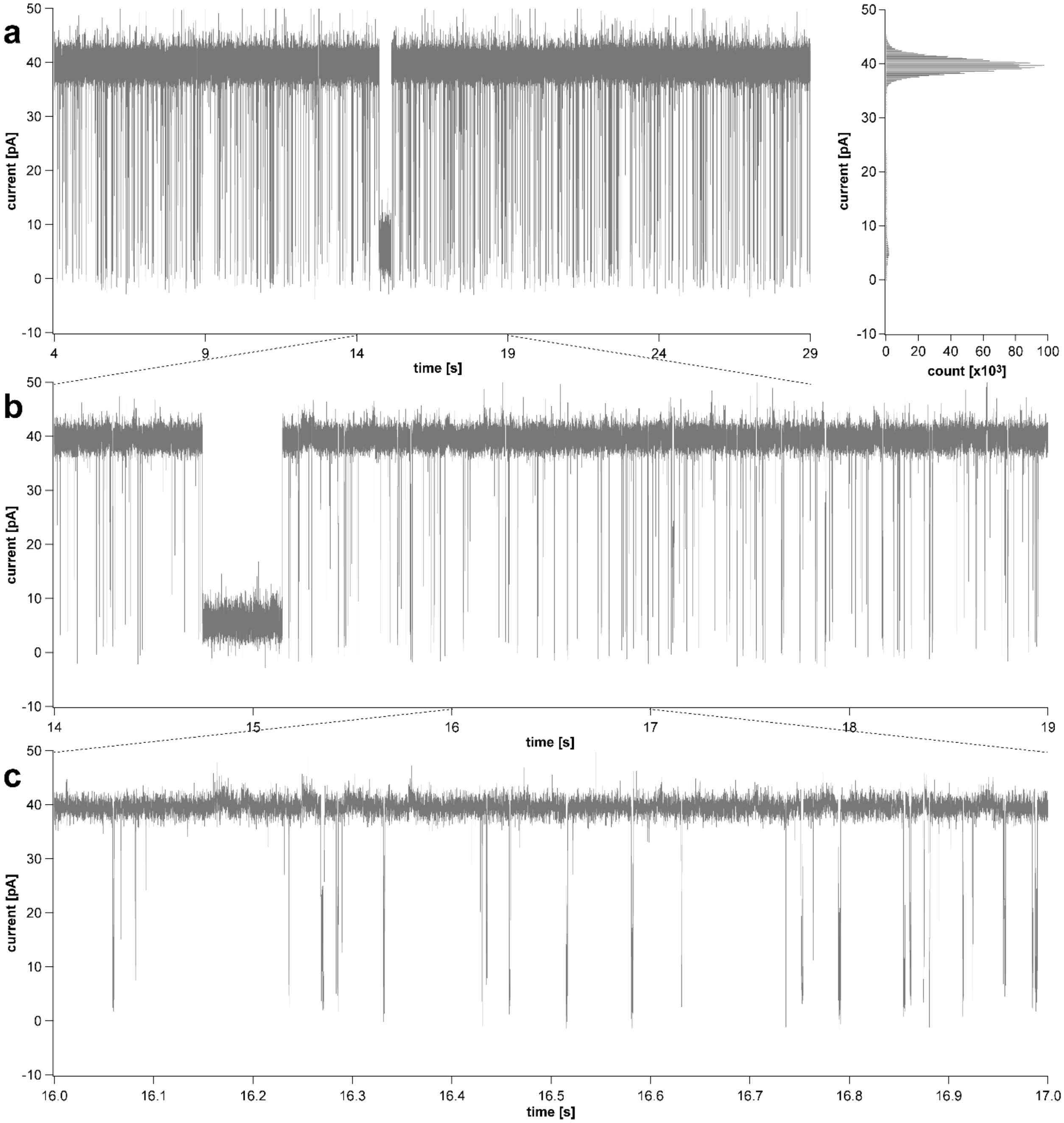

Figure S2): Representative single channel recording of OmpG in lipid bilayers at pH 7.6. a) 25 s segment of OmpG current trace and corresponding histogram. **b)** 5 s, and **c)** 1 s zoom-ins of current traces. Traces were recorded at +40 mV at 100 kHz sampling rate (and a 5 kHz electronic 4th order Bessel filter).

Supplementary Figure 3:

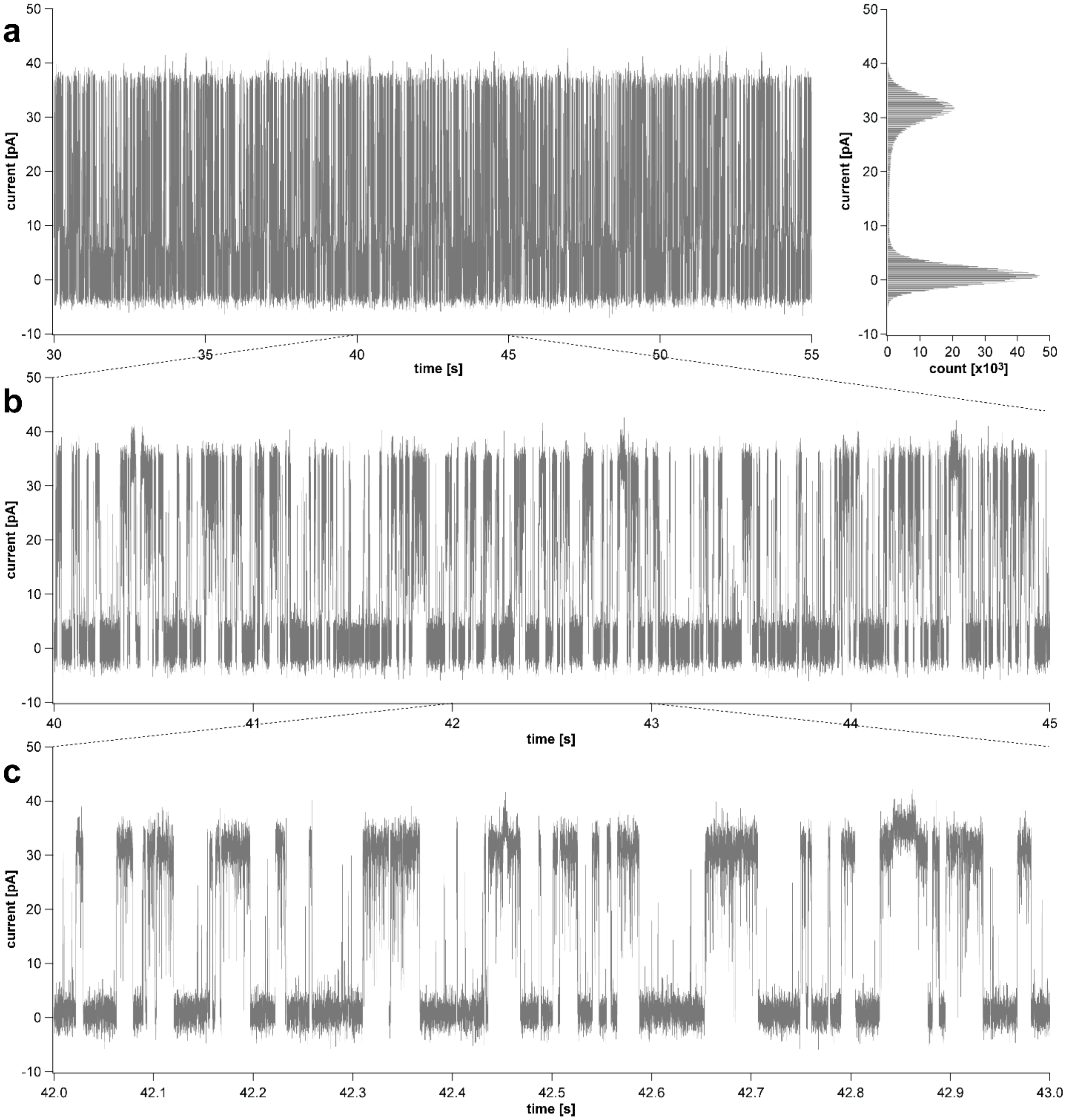

Figure S3): Representative single channel recordings of OmpG in lipid bilayers at pH 5.0. a) 25 s segment of OmpG current trace and corresponding histogram. **b)** 5 s, and **c)** 1 s zoom-ins of current traces. Traces were recorded at +40 mV at 100 kHz sampling rate (and a 5 kHz 4th order Bessel filter).

Supplementary Figure 4:

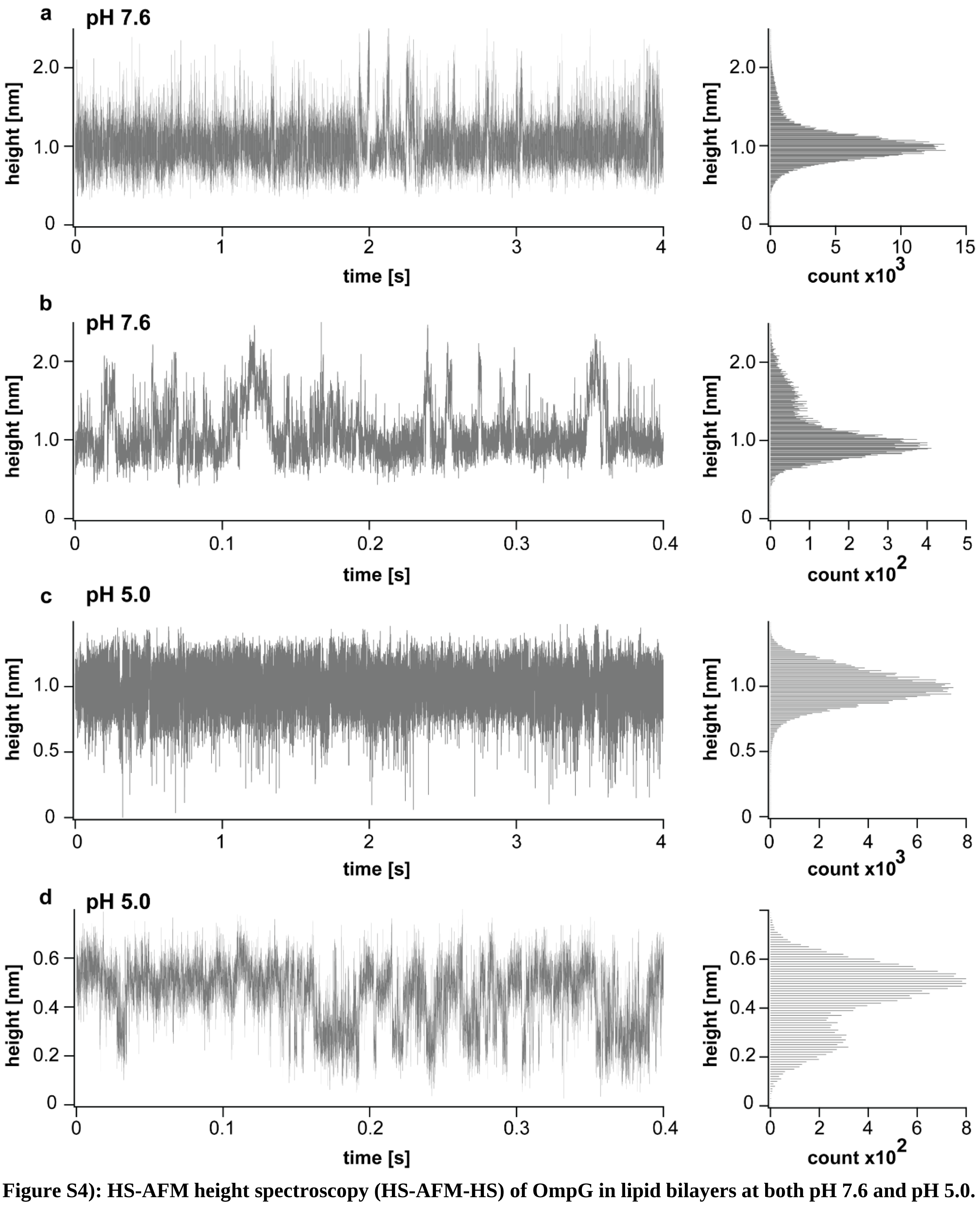

Figure S4): HS-AFM height spectroscopy (HS-AFM-HS) of OmpG in lipid bilayers at both pH 7.6 and pH 5.0. **a)** 4 s segment of OmpG height spectroscopy and corresponding histogram at pH 7.5. **b)** 0.4 s segment of OmpG height spectroscopy and corresponding histogram at pH 7.5. **c)** 4 s segment of OmpG height spectroscopy and corresponding histogram at pH 5.0. **d)** 0.4 s segment of OmpG height spectroscopy and corresponding histogram at pH 5.0.

Supplementary Figure 5:

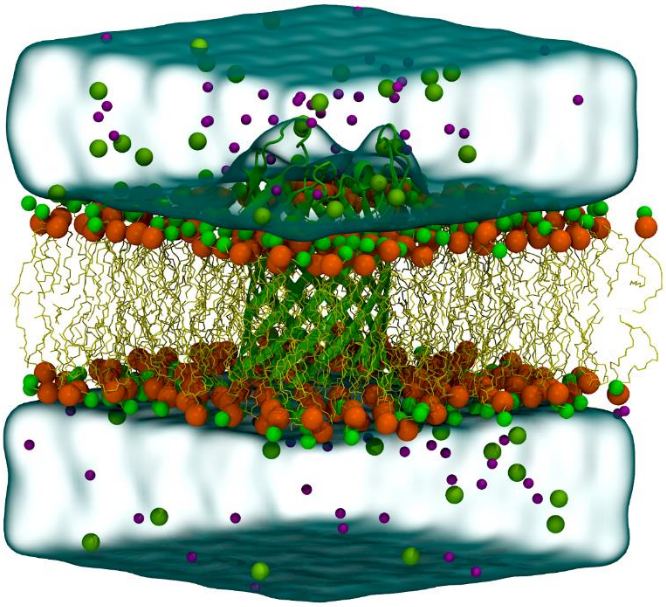

Figure S5) OmpG (Open state) in a fully hydrated dimyristoylphosphatidylcholine (DMPC) bilayer. The protein is shown in light green cartoon. Lipids units are depicted in yellow, while their phosphate and choline groups are illustrated as orange and green van der Waals spheres, respectively. Potassium and chloride counterions are shown in green and purple, respectively. A continuous and semi-transparent cyan representation is used for water.

Supplementary Figure 6:

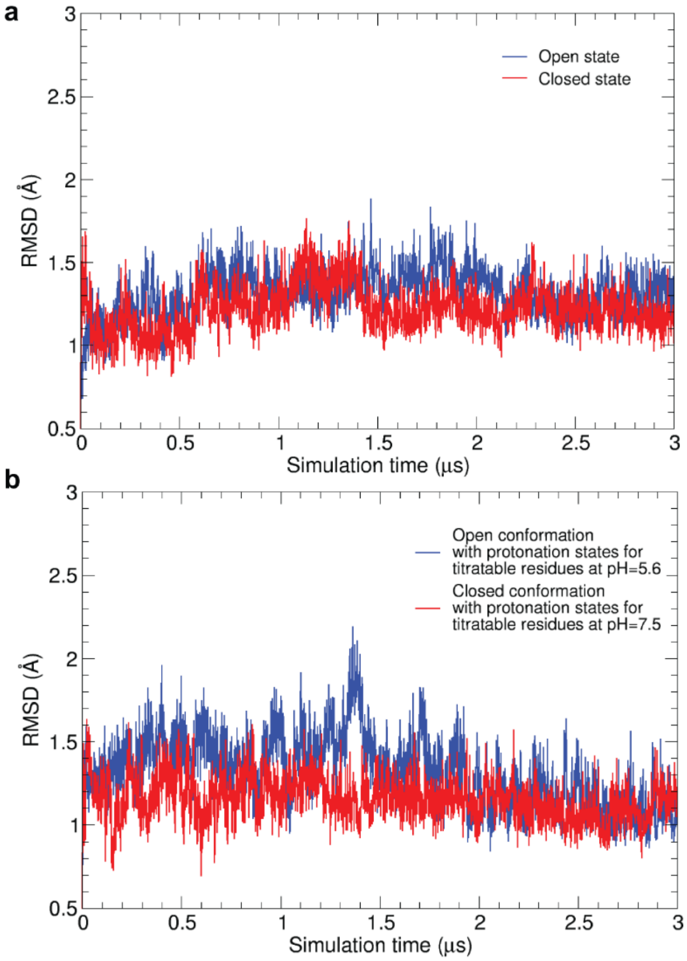

Figure S6): Time evolution of the RMSD over the backbone atoms of the β-barrel for OmpG in open and closed conformations. a) RMSD with respect to crystallographic geometries at corresponding pH values (PDB id: 2iwv and 2iww, respectively), and **b)** with swapped protonation states and using as reference the crystallographic geometries.

Supplementary Figure 7:

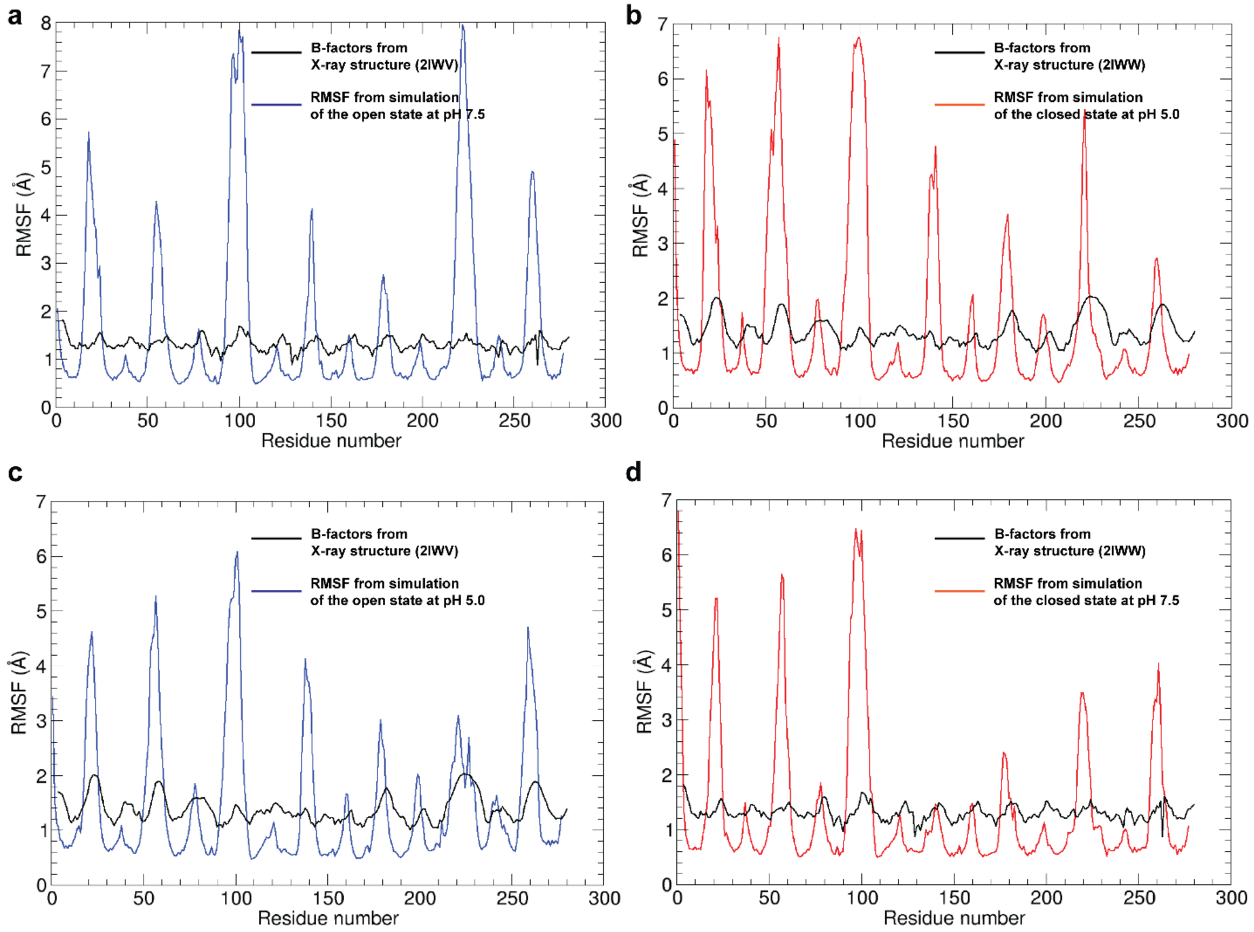

Figure S7) RMSF for residue Cα atoms of OmpG in open and closed conformations. a) B-factors (black) values from the crystallographic structures (PDB id: 2iwv) and RMSF values obtained from 3 µs simulation (blue) of open state at pH 7.5. **b)** B-factors (black) values from the crystallographic structures (PDB id: 2iww) and RMSF values obtained from 3 µs simulation (red) of open state at pH 5.0. **c)** B-factors (black) values from the crystallographic structures (PDB id: 2iwv) and RMSF values obtained from 3 µs simulation (blue) of open state at pH 5.0. **d)** B-factors (black) values from the crystallographic structures (PDB id: 2iww) and RMSF values obtained from 3 µs simulation (red) of closed state at pH 7.5.

Supplementary Figure 8:

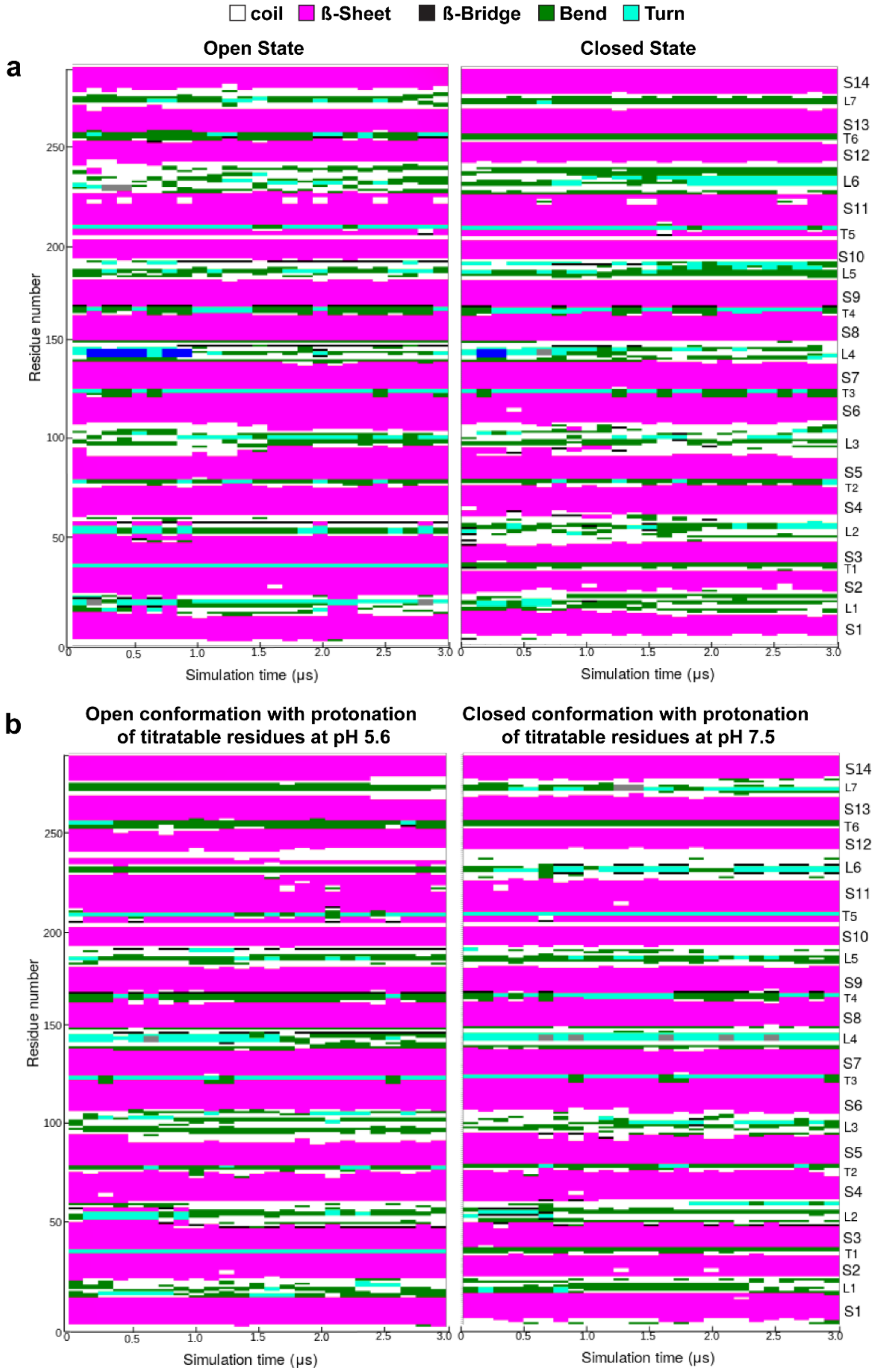

Figure S8) Time series of the secondary structure of OmpG. a) Open (left) and closed (right) stated. **b)** Open conformation at pH 5.0 (left) and closed conformation at pH 7.5 (right).

Supplementary Figure 9:

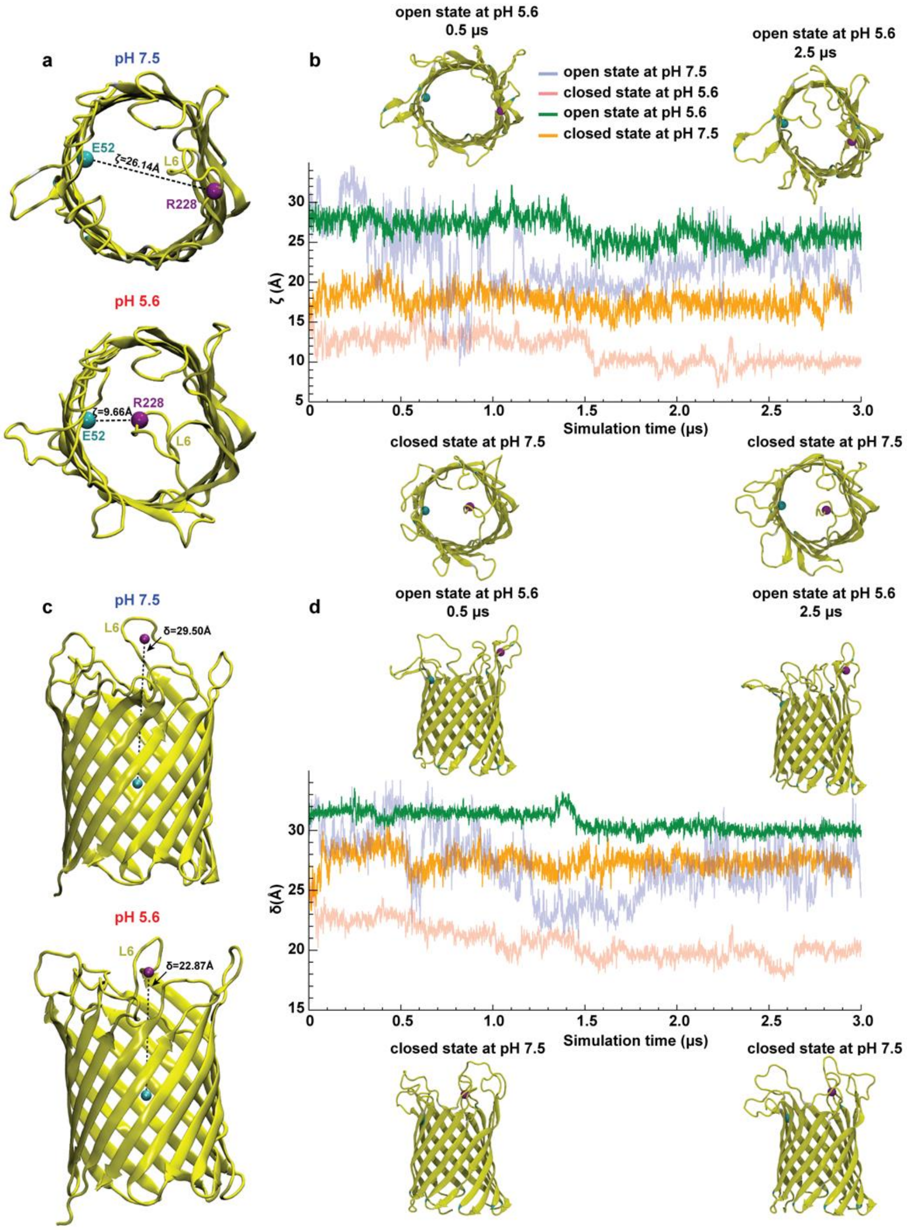

Figure S9) All atom molecular dynamics simulations of OmpG open and closed states in acidic and neutral conditions, respectively. a) and **b)** Collective variable ζ (the distance between the C_α atoms for the residues E52 (cyan spheres) and R228 (purple spheres)), and **c)** and **d)** collective variable δ (the distance between the centers of mass (COM) of the backbone atoms for the barrel (cyan balls) and the C_α atoms for residues 220 to 228 of loop-6 (purple spheres) during all atom MDS of the OmpG open and closed states in acidic and neutral conditions, respectively. Initial structural states and conditions are indicated above the traces in a). For comparison the traces of the open state at neutral pH and the closed state at acidic pH (from main manuscript Figure 3) are shown in semi-transparency. Representative structures at 0.5 and 2.5 µs are shown.

Supplementary Tables:

Supplementary Table 1:

	**pH 7.5**		**pH 5.0**	
Channel #	Open probability	Dwell time	Open probability	Dwell time
**1**	0.94	$<t_{open}> = 19.5$ ms<br>$<t_{closed}> = 1$ ms	0.35	$<t_{open}> = 2.93$ ms<br>$<t_{closed}> = 5.38$ ms
**2**	0.95	$<t_{open}> = 14.5$ ms<br>$<t_{closed}> = 0.75$ ms	0.47	$<t_{open}> = 1.6$ ms<br>$<t_{closed}> = 1.8$ ms
**3**	0.95	$<t_{open}> = 18$ ms<br>$<t_{closed}> = 0.93$ ms	0.30	$<t_{open}> = 1.24$ ms<br>$<t_{closed}> = 3$ ms
**4**	0.93	$<t_{open}> = 6.8$ ms<br>$<t_{closed}> = 0.43$ ms	0.40	$<t_{open}> = 2.5$ ms<br>$<t_{closed}> = 3.7$ ms
**5**	0.96	$<t_{open}> = 12.8$ ms<br>$<t_{closed}> = 0.5$ ms	-	-

Table S1: Open probabilities and dwell times of single channel electrophysiology recordings at neutral and acidic pH (short flickers to the closed state are not included in this table).

Supplementary Table 2:

	**pH 7.5**		**pH 5.0**	
Traces #	Low (open) probability	Dwell time	Low (open) probability	Dwell time
**1**	0.88	$<t_{open}> = 3.9$ ms<br>$<t_{closed}> = 0.6$ ms	0.30	$<t_{open}> = 0.8$ ms<br>$<t_{closed}> = 1.8$ ms
**2**	0.96	$<t_{open}> = 5.7$ ms<br>$<t_{closed}> = 0.2$ ms	0.27	$<t_{open}> = 0.6$ ms<br>$<t_{closed}> = 1.6$ ms
**3**	0.89	$<t_{open}> = 3.3$ ms<br>$<t_{closed}> = 0.4$ ms	0.46	$<t_{open}> = 2.1$ ms<br>$<t_{closed}> = 2.6$ ms
**4**	0.91	$<t_{open}> = 1.5$ ms<br>$<t_{closed}> = 0.15$ ms	-	-
**5**	0.85	$<t_{open}> = 1.4$ ms<br>$<t_{closed}> = 0.25$ ms	-	-

Table S2: Open probabilities of HS-AFM height spectroscopy experiments at neutral and acidic pH

Supplementary Table 3:

Residue	Sequence	Open structure (PDB: 2iwv); pH=7.5		Closed structure (PDB: 2iww); pH=5.6	
		*Predicted pKa	**Protonation state	*Predicted pKa	**Protonation state
GLU	15	5.53	Unprotonated	5.85	Protonated
GLU	17	4.34	Unprotonated	4.51	Unprotonated
GLU	20	3.37	Unprotonated	3.58	Unprotonated
GLU	24	5	Unprotonated	5.1	Unprotonated
GLU	31	10.32	Protonated	10.54	Protonated
GLU	52	6.16	Unprotonated	6.38	Protonated
GLU	70	3.6	Unprotonated	2.86	Unprotonated
GLU	72	4.57	Unprotonated	6.1	Protonated
GLU	79	4.68	Unprotonated	4.71	Unprotonated
GLU	101	4.23	Unprotonated	4.2	Unprotonated
GLU	152	4.28	Unprotonated	5.3	Unprotonated
GLU	154	4.1	Unprotonated	3.84	Unprotonated
GLU	163	3.98	Unprotonated	4.59	Unprotonated
GLU	174	5.62	Unprotonated	8.74	Protonated
GLU	187	4.44	Unprotonated	4.36	Unprotonated
GLU	192	3.92	Unprotonated	5.74	Protonated
GLU	227	4.63	Unprotonated	7.05	Protonated
GLU	229	6.97	Unprotonated	2.37	Unprotonated
GLU	253	5.19	Unprotonated	4.87	Unprotonated
GLU	257	4.49	Unprotonated	6.3	Protonated
GLU	263	5.06	Unprotonated	3.84	Unprotonated
HIS	7	6.27	HSD	6.44	HSP
HIS	74	6.47	HSE	5.82	HSE
HIS	97	6.1	HSE	6.22	HSP
HIS	204	6.43	HSE	6.14	HSP
HIS	231	4.81	HSD	9.57	HSP
HIS	261	6.84	HSE	7.14	HSP
HIS	270	6.83	HSD	6.85	HSP

Table S3. Computed pKa values and the protonation states used in the MD simulations for critical residues on OmpG. *Predicted values with propka 3.1. **Proton assignment based on the predicted pKa values at given pH. Protonation states for critical residues are highlighted in red.
