# Supplementary material for: Correlation of membrane protein conformational and functional dynamics
Source: Nat Commun. 2021 Jul 16;12:4363. doi: 10.1038/s41467-021-24660-1 (PMC8285522; doi:10.1038/s41467-021-24660-1)
Supplement: Supplementary file 3 — Description of Additional Supplementary Files [file 41467_2021_24660_MOESM3_ESM.pdf]

Description of Additional Supplementary Files

File Name: Supplementary Movie 1
Description: HS-AFM movies of OmpG in lipid bilayers at pH 7.6 (left) and pH 5.0 (right) recorded at 200ms per frame. Middle panel show average of OmpG dimers at both pH values.

File Name: Supplementary Movie 2
Description: Unbiased 3µs-long molecular dynamic simulation (MDS) of OmpG (side view) at physiological pH using crystal structure of the open state OmpG (PDB: 2iwv).

File Name: Supplementary Movie 3
Description: Unbiased 3µs-long molecular dynamic simulation (MDS) of OmpG (top view) at physiological pH using crystal structure of the open state OmpG (PDB: 2iwv).

File Name: Supplementary Movie 4
Description: Unbiased 3µs-long molecular dynamic simulation (MDS) of OmpG (side view) at acidic pH using crystal structure of the open state OmpG (PDB: 2iww).

File Name: Supplementary Movie 5
Description: Unbiased 3µs-long molecular dynamic simulation (MDS) of OmpG (top view) at acidic pH using crystal structure of the open state OmpG (PDB: 2iww).
