# Supplementary material for: Correlation of membrane protein conformational and functional dynamics
Source: Nat Commun. 2021 Jul 16;12:4363. doi: 10.1038/s41467-021-24660-1 (PMC8285522; doi:10.1038/s41467-021-24660-1)
Supplement: Supplementary file 9 — Reporting Summary [file 41467_2021_24660_MOESM9_ESM.pdf]

nature research

Corresponding author(s): Simon Scheuring

Last updated by author(s): Jun 3, 2021

Reporting Summary

Nature Research wishes to improve the reproducibility of the work that we publish. This form provides structure for consistency and transparency in reporting. For further information on Nature Research policies, see our Editorial Policies and the Editorial Policy Checklist.

Statistics

For all statistical analyses, confirm that the following items are present in the figure legend, table legend, main text, or Methods section.

n/a	Confirmed	
☐	☒	The exact sample size (*n*) for each experimental group/condition, given as a discrete number and unit of measurement
☐	☒	A statement on whether measurements were taken from distinct samples or whether the same sample was measured repeatedly
☒	☐	The statistical test(s) used AND whether they are one- or two-sided<br>*Only common tests should be described solely by name; describe more complex techniques in the Methods section.*
☒	☐	A description of all covariates tested
☒	☐	A description of any assumptions or corrections, such as tests of normality and adjustment for multiple comparisons
☐	☒	A full description of the statistical parameters including central tendency (e.g. means) or other basic estimates (e.g. regression coefficient) AND variation (e.g. standard deviation) or associated estimates of uncertainty (e.g. confidence intervals)
☒	☐	For null hypothesis testing, the test statistic (e.g. *F*, *t*, *r*) with confidence intervals, effect sizes, degrees of freedom and *P* value noted<br>*Give P values as exact values whenever suitable.*
☒	☐	For Bayesian analysis, information on the choice of priors and Markov chain Monte Carlo settings
☒	☐	For hierarchical and complex designs, identification of the appropriate level for tests and full reporting of outcomes
☒	☐	Estimates of effect sizes (e.g. Cohen's *d*, Pearson's *r*), indicating how they were calculated

Our web collection on statistics for biologists contains articles on many of the points above.

Software and code

Policy information about availability of computer code

Data collection	HS-AFM height spectroscopy data was captured with home written software and a data acquisition board (LabView programming, NI-USB-6366 card, National Instruments, USA). Single channel recordings were measured with Clampex (Molecular Devices) software. MD simulations: All-atom molecular dynamics (MD) simulations were performed with the NAMD program. pKa values of ionizable groups in proteins were predicted using program propka 3.1. Molecular interactions were described with the all-atom CHARMM36 force field for biomolecules. Long-range electrostatic interactions were evaluated using the particle mesh Ewald (PME) algorithm. RATTLE algorithm was employed to constrain the equilibrium length of all covalent bonds containing hydrogen atoms. In MD simulation the temperature and pressure were maintained at 298 K and 1 atm, respectively, using Langevin dynamics and the Langevin piston algorithm. The potentials of mean force (PMFs) were determined using a multiple-walker variant of the well-tempered metadynamics extended adaptive biasing force (WTM-eABF) algorithm.
Data analysis	HS-AFM height spectroscopy data was analyzed using self written routines in MATLAB (R2019b). To determine the states and state transitions, we adapted the Step Transition and State Identification (STaSI, Shuang et al, J Phys Chem Lett 2014) algorithm developed for discrete single-molecule data analysis for our HS-AFM-HS data. Single channel recordings were analyzed using clampfit 10.7 (Molecular Devices). All rendering and MD analyses were performed with the VMD version 1.9.3 software.

For manuscripts utilizing custom algorithms or software that are central to the research but not yet described in published literature, software must be made available to editors and reviewers. We strongly encourage code deposition in a community repository (e.g. GitHub). See the Nature Research guidelines for submitting code & software for further information.

Data

Policy information about availability of data

All manuscripts must include a data availability statement. This statement should provide the following information, where applicable:

- Accession codes, unique identifiers, or web links for publicly available datasets
- A list of figures that have associated raw data
- A description of any restrictions on data availability

Data supporting the findings of this manuscript are available from the corresponding author. Source data are provided with this paper.

Field-specific reporting

Please select the one below that is the best fit for your research. If you are not sure, read the appropriate sections before making your selection.

- [x] Life sciences
- [ ] Behavioural & social sciences
- [ ] Ecological, evolutionary & environmental sciences

For a reference copy of the document with all sections, see nature.com/documents/nr-reporting-summary-flat.pdf

Life sciences study design

All studies must disclose on these points even when the disclosure is negative.

Sample size	No statistical methods were used to determine sample size. Single channel currents and height spectroscopy measurements were recorded from at least 3 independent channels (as indicated).
Data exclusions	no data exclusions were made.
Replication	All data are representative of at least three independent bilayer preparations (3-5 independent single channel recordings) and two independent sample preparations for height spectroscopy (3-5 independent channel recordings) with results showing statistically similar results.
Randomization	Not applicable to this study as there was just one experimental group.
Blinding	Not applicable to this study as there was just one experimental group.

Reporting for specific materials, systems and methods

We require information from authors about some types of materials, experimental systems and methods used in many studies. Here, indicate whether each material, system or method listed is relevant to your study. If you are not sure if a list item applies to your research, read the appropriate section before selecting a response.

Materials & experimental systems

n/a	Involved in the study	
[x]	[ ]	Antibodies
[x]	[ ]	Eukaryotic cell lines
[x]	[ ]	Palaeontology and archaeology
[x]	[ ]	Animals and other organisms
[x]	[ ]	Human research participants
[x]	[ ]	Clinical data
[x]	[ ]	Dual use research of concern

Methods

n/a	Involved in the study	
[x]	[ ]	ChIP-seq
[x]	[ ]	Flow cytometry
[x]	[ ]	MRI-based neuroimaging
